# Factors determining implicit attitudes toward sports and exercise: desirability and enjoyment

**DOI:** 10.1007/s44202-023-00073-7

**Published:** 2023-04-20

**Authors:** Megumi M. Ohashi, Takafumi Sawaumi, Yumiko Iume, Etsuko Togo

**Affiliations:** 1 Faculty of Child Psychology, Tokyo Future University, Senjuakebono-Cho, Adachi-Ku, Tokyo, 120-0023 Japan; 2 Faculty of Sociology, Ryutsu Keizai University, 3-2-1 Shin-Matsudo, Matsudo-Shi, Chiba, 270-8555 Japan

**Keywords:** Implicit attitudes, Implicit Association Test, Dual attitude perspective

## Abstract

Previous studies have found that implicit attitudes, in addition to explicit attitudes, toward sports and exercise can help predict health-related behaviors. This study aimed to explore the factors that determine implicit attitudes toward sports and exercise. Using an online experiment, we investigated two types of implicit attitudes toward sports and exercise: desirability and enjoyment. Implicit attitudes toward sports and exercise were measured using two different Implicit Association Test (IAT) for desirability and for joy. We examined the degree to which “sports and exercise” were perceived to be more strongly associated with positive words than “sedentary behaviors.” We hypothesized that past experiences with sports and exercise affect implicit attitudes, and that desirability and enjoyment (positive implicit attitudes) might differ qualitatively. Participants included 318 students (230 male and 88 female, *M* = 19.62, *SD* = 1.78) who completed online questionnaires consisting of one of the two types of IAT. The results indicated that male participants have more positive implicit attitudes for both joy and desirability, and people with sports competence tend to have higher positive implicit attitudes concerning joy, but not desirability. Interest in professional sports was related to both IATs, while only the joy IAT was related to self-reported physical competence and the frequency of playing sports. The contributions of this study show that the two types of implicit attitudes toward sports and exercise—desirability and joy—are associated with different behaviors, and perceiving sports and exercise as joyful leads people to lifelong sports participation.

## Introduction

It is a well-established fact that sports and exercise are beneficial for maintaining health [1, 2]. In addition, physical exercise and sports have been shown to activate the brain [3] and improve mental health [4]. In recent years, health problems owing to physical inactivity have become a crucial issue in many countries. Although the World Health Organization (WHO) has issued global guidelines to indicate the amount of physical activity required for each age group, many people do not meet these standards [5, 6]. This may be owing to the abundance of electronic devices and convenient lifestyles that restrict physical activity [7].

While a fair number of children participate in sports and exercise through extracurricular club activities at school, the participation rate declines with age [8, 9]. In Japan, where sports is a popular extracurricular school activity, many students stop engaging in regular sports and exercise upon graduation from junior high or high school. Those who continue to participate in sports and exercise after entering the workforce are a minority, at around 20% [10, 11]. However, some adults do continue to play some form of sports. In this study, we focus on the factors which contribute to the difference in participation in sports and exercise.

### Determinants of the practice of sports and exercise

According to a WHO report, 17% of people in the world are inactive [12], which means that they have a health risk. Physical inactivity tend to increase blood pressure and cholesterol, which raises the risk of chronic diseases such as heart disease, diabetes and cancers [12]. Several studies have examined the reasons people do or do not participate in these activities and have shown that time and financial freedom, peers to perform activities with, social pressure, and environmental ease increase the probability of engagement in sports and exercise [13, 14]. However, despite the presence of these environmental factors, some individuals report that they do not engage in exercise for unspecified reasons [11]. A common explanation is that psychological factors, such as attitude toward sports and exercise, may also impact people’s willingness to participate in these activities. Van Stralen et al. [15] reviewed many studies on the factors that initiated and led to continuity in sports and exercise. They showed that the recognition of the physical and psychological positive aspects of sports and exercise significantly increased the long-term commitment to such activities [15].

In general, the more desirable an object is perceived to be, the stronger the approach motivation is toward it [16]. Applying the same reasoning to sports and exercise, it could be speculated that the more pleasant or interesting people think sports and exercise are, the more likely they are to engage in them willingly. In other words, having an enjoyable and pleasant perception of healthy habits (i.e., exercise, dietary restrictions), in addition to placing a utilitarian value on them, such as considering them important or useful, influences the extent that individuals exercise or follow dietary restrictions [17]. Therefore, it is necessary to consider how positive attitudes toward sports and exercise can be formed from the perspective of lifelong sports promotion.

Although the terms “sports” and “exercise” are originally different, their meaning overlaps. In a survey of Japanese adults, approximately half answered that the two meant the same [18]. Additionally, prior research has not always examined sports and exercise separately. For example, a survey conducted by the Sasagawa Sports Foundation [19] asked questions that combined the two (e.g., “How often do you play sports and exercise?”). Therefore, in this study, we examined attitudes toward sports and exercise without separating the terms.

### Attitudes toward sports and exercise

In psychology, attitudes are defined as “a psychological tendency that is expressed by evaluating a particular entity with some degree of favor or disfavor” [16]. Attitudes, operating as a form of psychological readiness in which an individual decides how to react or behave toward a particular entity, are often examined using self-report methods [16]. As for sports and exercise, several studies employ self-reports to measure attitudes toward sports and exercise [14, 15, 17, 19]. However, since the self-report method allows participants to choose what to communicate themselves, bias is unavoidable. For instance, participants may respond favorably because sports are socially desirable.

However, in recent years, implicit attitudes (attitudes without introspective processes) have been measured using reaction times. According to dual-process theories [20], behavior is motivated by both explicit (i.e., controlled) and implicit (i.e., automatic) processes. That is, automatic processes and behaviors reflect implicit attitudes whereas intentional processes and actions reflect explicit attitudes. Implicit attitudes are often not aligned with explicit ones and have their own predictive power for behavior and other related variables [21].

Both implicit and explicit attitudes toward sports and exercise influence behavior and behavioral intentions. For example, Conroy et al. [22] and Hyde et al. [23] used the Implicit Association Test (IAT) to examine physical activities—a method that indirectly measures the strength of association among concepts using reaction time [24]. Both of them showed that implicit attitudes toward sports and exercise positively predicted related behavior. In particular, they demonstrated that implicit attitudes positively predicted less-intentional physical activity in the form of daily steps, after controlling for the positive effect of explicit attitudes. In Hyde et al. [23], implicit attitudes did not directly predict self-reported physical activities nor daily steps; however, the change in implicit attitudes positively predicted these activities.

### Purpose of the current study

The current study aimed to explore the factors that determine implicit attitudes toward sports and exercise. Adolescence and young adulthood are important periods for the formation of various attitudes. The belongingness to a sports organization and the way coaches treat their players, can have a significant impact on players’ attitudes [25]. We assumed that sports-related experiences in youth, especially club activities, may have a significant influence on implicit and explicit attitudes toward sports and exercise. It is said that educational instructors, or coaches, provide two types of motivational atmospheres [26]. One is task-involved instruction that focus on learning and progress, such as “encouraging students to work hard,” and the other is ego-involved instruction that focuses on each performance and achievement, such as “considering only highly capable and talented students as important individuals.”[26] These are applicable to sports. Previous studies have suggested that only a task-involved climate increases the perceived autonomy, competence, relatedness, intrinsic motivation, and future intention to keep playing [27]. Another is experience of harassment by coaches. Sports activities sometimes involve harassment [28, 29]. Togo and her colleges suggest that experiencing sports harassment from coaches can lead to decreased motivation for sports and increased neurotic tendencies [30]. We then hypothesized that the experience of joining a sports club and task-involved coaching styles would have a positive effect, and that the experience of sports harassment and ego-involved coaching styles would have a negative effect on attitudes toward sports and exercise (Hypothesis 1).

We also considered the types of attitudes. Previous studies have shown that affective and cognitive attitudes differ [31]. In the context of sports and exercise, for example, Experiment 1 in Trafimow and Sheeran [32], which measured attitudes toward smoking behavior, revealed that affective attitudes had a greater influence on smokers' behavioral intentions than cognitive attitudes. Furthermore, Experiment 2, which assessed attitudes toward studying, measured how much the students studied during the winter break and showed that cognitive attitudes had a greater influence on behavior than affective attitudes. Another study showed that affective factors had a more direct effect on intentions regarding health behaviors, including exercise, after statistically controlling for past behavior, suggesting that affective attitudes may promote more voluntary and unintentional behavior [33].

Similarly, it can be assumed that people hold both cognitive and affective attitudes toward sports and exercise. For example, an individual may acknowledge that sports and exercise are important for staying healthy and maintaining one’s shape (cognitive attitude, related with evaluative aspects, such as usefulness or desirability) but still not like these activities (affective attitude, related with intuitive and emotional aspects, such as enjoyment or satisfaction). Therefore, we considered two types of implicit and explicit attitudes toward sports and exercise: the degree of desirability and the degree of enjoyment. We hypothesized that the degree of enjoyment is more strongly related to current sports and exercise behaviors, than the degree of desirability, even for implicit attitudes (Hypothesis 2).

To test our hypothesis, university students were recruited to participate in an online study. The first half of the study included an IAT task [24] on sports and exercise and the second half consisted of answering questions. The participants were given one of the two types of IATs (desirability IAT or joy IAT).

## Methods

### Participants

Students from psychology classes at three universities in the Tokyo metropolitan area were recruited to participate in the study (*N* = 323) from late November 2020 to early January 2021. In current study, the average correct response rate was 93.75% (*SD* = 6.11). We judged the participants with under *M*–3*SD* (75.4%) correct rate to be “extremely high error rate.” We excluded two participants with extremely high error rate, in addition to three who dropped out of the study. As a result, 318 participants (230 male and 88 female students) were included in the final analysis. Their mean age was 19.62 years (*SD* = 1.78), except for three individuals whose age was unknown.

All instructions were provided in Japanese. Five students were from China/Taiwan, two were from other Asian countries, and one was from Europe. As their classes were conducted in Japanese and the research data showed no problems with reaction time, all participants were judged as having sufficient Japanese language ability. The analysis with only Japanese participants showed the same patterns.

Before the study began, participants were informed that participation was voluntary, anonymity was ensured, and that the results which would be published, were unrelated to their class performance. This study was conducted with the approval of the research ethics committee of the Tokyo Future University, Japan (no. 2020-11).

### Design

Independent variables comprised the type of attitudes (desirability or joy, a between-participants factor) and the measurement style (implicit or explicit, a within participants factor). The dependent variable was attitude toward sports and exercise. The type of attitude was adopted as a between-participants factor to not overburden the participants by making them perform two similar tasks. A total of 148 were assigned to Desirability IAT condition, and 170 were assigned to Joy IAT condition.

### Procedure

This study was conducted online. The title is “An Experiment on the Values and Attitudes Held by College Students Toward Sports and Exercise.” We requested students enrolled in introductory psychology classes at three private universities to participate in the study. All participants who accessed the research site were randomly assigned to the Desirability IAT condition or the Joy IAT condition.

The participants were asked for information regarding their age, gender, and major. Next, an IAT task, called the word classification task, was administered to measure implicit attitudes toward sports and exercise. We used conventional IAT procedure developed by Greenwald and colleagues [24], using Inquisit 6 (millisecond, Seattle, WA, USA) and personal computers. Then, there were questions regarding participants’ experience of sports and exercise in school, followed by the measurement of explicit attitudes toward sports and exercise. Finally, a debriefing session was conducted. It took about 20–25 min for the entire experiment.

### Measured variables

#### Implicit attitudes toward sports and exercise

Implicit attitudes were measured using the conventional IAT procedure developed by Greenwald and colleagues [24]. This test measures implicit (hidden, unconscious) associations between categories. Participants were asked to classify a word shown in the center of the computer screen into one of the four categories, one by one. They were instructed to react as quickly and correctly as they could. The more strongly the two categories on the same side of the screen were linked in the participants’ minds, the more easily and quickly they could categorize the words. In such cases, the response times were shorter.

The target category was “sports and exercise.” The conventional IAT requires a category that opposes the target category; for this, we used “sedentary behaviors.” The IAT also requires both positive and negative categories, and we used categories labeled “positive” and “negative.” In the context of this study, “conditions” means the types of attitudes, i.e., joy, or desirability among participants. The difference between the two conditions were words used in the IAT task (Table 1). The desirability IAT condition used words related to desirability, such as beneficial and bad. In the joy IAT condition, words related to enjoyment, such as happy and sad were used. The category labels were the same, “positive” and “negative.”

**Table 1:** Words used in the IAT

Kind of IAT	Category	Words
Target	Sports and exercise	Running, soccer, physical strength, cycling, muscle
	Sedentary behaviors	Having tea, reading, sofa, smartphone, recliner chair
Attribute: desirability	Positive	Important, beneficial, desirable, useful, splendid
	Negative	Terrible, stupid, incompetent, useless, bad
Attribute: joy	Positive	Happy, bright, excited, nice, wonderful
	Negative	Sad, miserable, hard, terrifying, painful

*IAT* implicit association test

The stimulus words were selected from a preliminary survey conducted prior to this study with 31 college students living in the Tokyo metropolitan area (23 female and 8 male students,* M* = 20.97 years, *SD* = 1.20; 21 had experience with sports clubs in junior high or high school). The candidate words in the preliminary survey were those listed as “physical activity” and as “sedentary behavior” in Chevance et al. [34]; “having tea,” “smartphones,” and “games” were also added as sedentary behaviors after discussion with several university students. For each of the 26 words, we asked, “Do you think ‘XX’ is close to ‘sports/exercise’?” Responses were given on a six-point scale. Consequently, five words were selected which had a mean value of 5.5 or higher for “sports/exercise,” and five words were selected from those with a mean value of 2.5 or lower for “sedentary behaviors” (Table 1). Since many words were evaluated as being close to “sports/exercise,” the content was thus balanced carefully, for example, by not using only the names of events.

Participants were asked to classify 240 words in trial sessions, and 40 words in each of the test blocks. The order of the consistent (i.e., positive and sports/exercise on the same side) and inconsistent (i.e., negative and sports/exercise on the same side) blocks was counterbalanced. The IAT D-score was calculated by subtracting the average of the response time of the consistent block from the average of the response time of the inconsistent block, and dividing by the aggregated *SD*. Among several variations in D-score calculation, we used D_1_ from Greenwald et al. [35].

#### Sports experience in junior high and high school

First, participants were asked, “Did you participate in any sports-related club activities or lessons for more than one year in junior or senior high school?” The responses were “yes” or “no.” Those who answered “no” were asked to choose “not applicable” and their responses were treated as missing values for the following questions.

Those who answered “yes” were asked a series of sport-related open questions such as the name of the club activity or the type of sport they engaged. We also inquired about the place of activity, by asking “Was it a school club activity?” and participants chose from “yes (junior high school and high school),” “yes (junior high school only),” “yes (high school only),” or “no.”

Regarding the duration of the activity, they were asked, “How long did you play it for?” This response was open-ended. They were also asked to indicate the importance of the sports-related club activities, using a five-point scale from “important (5)” to “not important at all (1).”

Participants also indicated the competition level of their club or team using a six-point scale: “national tournament level (6),” “regional tournament level (5),” “prefectural tournament level (4),” “high district tournament level (3),” “mid-district tournament level (2),” and “lower district tournament level (1).” Their personal competition level was also measured using a five-point scale from “good (5)” to “poor (1).”

Two scales were used to measure instructors’ coaching style. First is the Motivational Atmosphere in Sports scale [36], which measures the instructor's involvement style. It measures task-involved instruction that focuses on achievement goals, such as “encouraging students to work hard,” and ego-involved instruction that focuses on performance goals, such as “considering only highly capable and talented students as important individuals.” The original scale consists of 12 items. We used three items for each factor in descending order of factor loadings, because using too many items can be burdensome for the respondents.

Next, we measured participants’ experience of harassment by coaches using a nine-item scale [37]. This scale assesses the frequency of harassment by coaches with items such as “Coach (supervisor, adviser) yelled at us more than necessary” and “Coach (supervisor, adviser) hit us when they became excited.” The items were rated on a six-point scale: every time (6), once or twice a week (5), once or twice a month (4), four or five times a year (3), once or twice a year (2), and never (1).

The subsequent questions were posed to all participants.

#### Explicit attitudes toward sports and exercise

After the leading phrase, “For you, sports and exercise is…,” the words “enjoyable,” “pleasant,” “satisfying,” “important,” “valuable,” and “useful” were inserted, and the participants were asked to rate each of these six statements using a seven-point scale, from “agree very much (7)” to “do not agree at all (1).” The first three items were intended to measure explicit attitudes concerning enjoyment, whereas the last three items measured explicit attitudes concerning desirability. The items have been used in previous studies on attitudes toward sports and exercise [17, 32].

#### Behaviors related to sports and exercise

Participants were asked to rate the items in Table 2, which were designed based on the criterion of daily behaviors that might be associated with either the desirability or joy of sports and exercise. For each item, responses were given on a four-point scale: yes (4), somewhat yes (3), somewhat no (2), and no (1).

**Table 2:** Items of daily behaviors related to sports and/or exercise (factor analysis)

Item		*M*	*(SD)*	Factor loading				Communality
				F1	F2	F3	F4	
F1. Contact behaviors (*M* = 1.94, *SD* = 0.95, *α* = .91)								
Do you have a favorite athlete?		2.01	(1.19)	**.96**	− .09	.04	− .03	.86
Are there any athletes you strongly support?		1.96	(1.25)	**.91**	− .13	.10	− .04	.76
Do you often check sports news?		2.15	(1.18)	**.83**	.20	− .12	.05	.84
Do you often watch sports? (Including on TV)		2.35	(1.17)	**.82**	.16	− .14	.06	.78
Do you often buy sports magazines?		1.22	(0.62)	**.47**	− .08	.29	− .02	.33
F2. Liking (*M* = 2.62, *SD* = 0.87, *r* = .48, *p* < .001)								
Do you like exercising and playing sports?		3.16	(0.86)	.00	**.74**	.01	.01	.54
Do you wear clothes made by sports brands?		2.07	(1.15)	.16	**.49**	.15	− .06	.46
F3. Health disregard (*M* = 2.06, *SD* = 0.75,* r* = .43, *p* < .001)								
Are you familiar with what is good for health?		2.14	(0.95)	.02	.13	− **.80**	− .07	.72
Do you care about your diet?		2.29	(0.97)	− .07	.04	− **.42**	.35	.41
F4. Weight and shape (*M* = 2.54, *SD* = 0.96, *r* = .63, *p* < .001)								
Do you care about your weight?		2.54	(1.12)	.07	− .17	− .01	**.83**	.66
Are you careful about your body shape?		2.53	(1.01)	− .05	.17	.04	**.78**	.68
Factor contribution				4.02	2.61	2.10	1.74	
Inter-factor correlation		F2		.57				
		F3		− .35	− .45			
		F4		− .01	− .31	− 0.43		

*M* mean, *SD* standard deviation, *F* = factor

#### Engaging in sports or exercises

Participants were enquired about their current sports habits as a college student. First, the frequency of actual sports and exercise activities was assessed as follows: “How many days a week have you engaged in sports and exercise on average during the past year? (Including jogging and training for more than 30 min)”; participants responded on a six-point scale: 5 or more days a week (6), 3 − 4 days a week (5), about 2 days a week (4), about 1 day a week (3), less than once a week (2), and not at all (1). This item was taken from the Japan Sports Agency’s “Public Opinion Survey on the State of Sports Implementation” [11].

The ideal frequency of sports and exercise activities was also measured because we believed that in some cases, owing to the COVID-19 pandemic in 2020 (when the study was conducted) and other environmental factors, people may have wanted to engage in sports and exercise but were unable to do so. Thus, we asked, “If the environment permitted, how many days a week would you be willing to engage in sports/exercise?” We used the same six-point scale as in the previous question.

#### Perceived physical competence

Finally, we assessed whether the participants were good at sports and exercise or not using four of the 12 items of the Physical Competence Scale [38]. Perceived physical competence was measured because in general, the better individuals perform an activity, the more enjoyment they experience, and the more motivated they are [36]. With regard to sports and exercise, we considered that physical competence was more likely to be evident in physical education classes, and that the perceptions of physical competence formed there were likely to influence individuals’ behavior in adulthood.

This scale includes three subscales: perception of physical competence, which indicates a positive perception of one’s athletic ability and physical skills; sense of control, which indicates the degree that a person perceives that they can control sports-related performance through effort and practice; and sense of acceptance, which indicates acceptance by teachers and peers concerning physical competence. In this study, we utilized only four items related to the perception of physical competence. The participants were asked to respond to questions such as, “I think I have excellent physical skills” and “I am confident in my physical skills,” using a five-point scale ranging from “5” (often true) to “1” (not at all true).

### Analysis

The analysis conducted in this study consisted of several statistical techniques. Firstly, a frequency and descriptive analysis was performed to examine the distribution of the variable, followed by a t-test to determine if the scores for the IAT were significantly higher than 0.

Secondly, an exploratory two-way ANOVA was conducted. This analysis aimed to investigate the potential differences between joy and desirability IATs and their respective impact on the results.

Thirdly, a factor analysis was performed on explicit attitudes items to determine if joy and desirability could be treated as different or not. We also did Pearson’s correlation analysis between explicit attitudes, the joy and desirability.

Fourthly, a factor analysis of sport and exercise behavior was performed to identify underlying factors that could explain the relationship among these variables.

Fifthly, Pearson’s correlation analysis was conducted between IAT scores and various scales such as sports experience in junior and senior high school, explicit attitudes, behaviors related to sports and exercise, engaging in sports or exercises, and perceived physical competence. This analysis aimed to investigate the relationship between implicit attitudes about sports and exercise and various variables related to sports and exercise, partly examining hypothesis 1.

Finally, a multiple regression analysis with the behaviors related to sports and exercise as predictor variables was conducted to examine hypothesis 2.

We analyzed the desirability IAT and joy IAT separately, in case interaction effects existed. Correlational analysis with behaviors related to sports/exercise and attitudes was also performed for exploratory purposes. The SPSS ver.27 was used for the analysis.

## Results

### Distribution of sports and exercise experience

A total of 259 (81.45%) had participated in sports-related club activities or lessons for at least one year during junior or senior high school. The male participation rate (87.39%) was significantly higher than the female participation rate [65.91%; *χ*^2^(1) = 19.44, *V* = .25, *p* < .001].

Most participants (78.93%) chose school club activities for participation in sports and physical activities. Among them, 52.02% of the respondents participated in individual sports and 47.98% in team sports. The responses to importance of the activity did not show a normal distribution, with “important” and “somewhat important” accounting for 61.01%, with a median of 4 (somewhat important).

The competition level of the teams had a skewed distribution in a more low-ranked form (skewness = 0.45): national tournament level (6, 5.97%), regional tournament level (5, 6.92%), prefectural tournament level (4, 13.84%), high district tournament level (3, 14.78%), middle district tournament level (2, 16.67%), and low district tournament level (1, 19.81%); the median value was three. In contrast, the distribution of individual competition levels approximated a normal distribution (skewness = -0.21, *M* = 3.23, *SD* = 1.19).

The internal consistency coefficient alpha of the Motivational Atmosphere in Sports scale was sufficiently high (α = .73 for task-involved instruction and α = .78 for ego-involved instruction). Therefore, the mean of the related items was used as the index (*M* = 3.56, *SD* = 0.85 and *M* = 2.62, *SD* = 1.00, respectively). Their skewness values were − 0.55 and 0.24. The internal consistency of the Coaching Harassment Scale was also high (α = .83); therefore, the mean of the nine items was used as the index (*M* = 1.37, *SD* = 0.68). Its distribution was highly skewed in a more low-ranked form (skewness = 2.73).

High internal consistency was observed for the perceived physical competence subscale of the Physical Competence Scale (α = .86). Therefore, the mean of the four items was used as the index (*M* = 2.94, *SD* = 1.05).

### Implicit attitudes toward sports and exercise

Participants’ IAT scores ranged from − 1.33 to 1.72, with a mean of 0.52 (*SD* = 0.55), which was significantly higher than 0 [*t*(317) = 16.84, *p* < .001]. After removing data with fewer than 70% correct responses, the mean correct response rate was 93.79% (*SD* = 5.77).

Next, a two-factor analysis of variance was conducted using IAT type (2) and participants’ gender (2) as independent variables and IAT scores as the dependent variable. As the order of the test sessions (consistent first or inconsistent first) was not a significant factor, we excluded the order effect in the following analyses. The results showed a significant main effect of gender [*F*(1,314) = 16.45, *η*_*p*_^2^ = .05,* p* < .001], with male students scoring significantly higher than female students (*M* = 0.60, *SD* = 0.49 vs. *M* = 0.32, *SD* = 0.64). No other main or interaction effect was observed. The main effect of IAT type [*F*(1,314) = 0.13, *η*_*p*_^2^ = .00,* p* = .72] and the interaction effect *F*(1,314) = 2.05, *η*_*p*_^2^ = .01,* p* = .15) were not significant.

### Explicit attitudes toward sports and exercise

Internal consistency was high for the desirability of sports and exercise (α = .91), and the mean value of these three items was used as the index (*M* = 5.49, *SD* = 1.22). The internal consistency coefficient was also high for the joy of sports and exercise (α = .91), and the mean value of these three items was used as the index (*M* = 5.51, *SD* = 1.22).

A fairly high correlation was found between the two variables [*r*(316) = .82, *p* < .001]. However, a factor analysis (maximum likelihood method, promax rotation) showed that the two-factor solution (CFI = .99, AIC = 30.09, RMSEA = .06) was a better fit than the one-factor solution (CFI = .95, AIC = 95.36, RMSEA = .16); therefore, as planned, they were treated as separate variables.

### Sports and exercise-related behaviors

Factor analysis (promax rotation with principal factor method) was conducted on sports- and exercise-related behaviors. Two items with commonalities less than 0.20 were excluded in the preliminary analysis. Four factors were obtained based on the eigenvalue criterion and interpretability of more than one; however, one item with a factor loading less than .40 was excluded, and the results were reanalyzed (Table 2).

Factor 1 was named “contact behaviors” because it was associated with having a favorite athlete and checking sports-related news. Factor 2 was named “liking” as it was associated with wanting to play sports and exercising and wearing clothes made by sports brands. Factor 3 was named “health disregard” because it indicated a tendency to neglect or ignore things that are good for the body, including diet. Factor 4 was named “weight and shape” as it was related to the degree of concern about one’s weight and shape. Some correlations were observed between these factors. Subscale scores were obtained by averaging the scores of items with factor loadings of .40 or higher.

The median actual frequency of sports and exercise was approximately one day per week (3), and the mode was less than once per week (2). The ideal frequency of sports and exercise was strongly positively correlated with the actual frequency of sports and exercise (*r*(316) = .62, *p* < .001), with a median of “about 2 days per week (4)” and a mode of “3 − 4 days per week (5).”

### Hypothesis testing

The correlations between IAT scores and various scales are summarized in Table 3. The correlation coefficients reported were calculated using Pearson's product-rate correlation coefficients. In cases such as when the distribution was skewed, the use of this coefficient was not statistically correct, and Kendall’s rank correlation coefficient was reported. Weak positive correlations were found between IAT scores and the two types of explicit attitudes measured using self-reported methods. Moreover, IAT scores were positively and weakly correlated with contact behavior and liking. A weak positive relationship between IAT scores and ideal frequency of sports and exercise was found only in the desirability condition, whereas a positive correlation with the actual frequency of exercise was found only in the joy condition. In addition, positive correlations between perceived physical competence and IAT scores were found only in the joy condition. Sports experience in high school had little effect, and a weak positive correlation was observed between the experience of task-oriented instruction and IAT scores in the joy condition.

**Table 3:** Correlations with the IAT score

	Desirability IAT	Joy IAT
Frequency of sports and/or exercise		
In reality (rank correlation coefficient)	.07	.19*
Ideal (rank correlation coefficient)	.17*	**.10**
Daily behaviors related with sports and/or exercise		
Contact behaviors	.28**	.18*
Liking	.19*	.19*
Health disregard	.03	.10
Weight and shape	− .01	− .01
Perceived physical competence	.08	.23**
Explicit attitudes: Desirable	.22**	.17*
Explicit attitudes: Joy	.18*	.24**
Sports experiences		
Personal competition level	.10	.13
Competition level of the club (rank correlation coefficient)	.03	− .04
Ego-involving motivational climate	.05	.00
Task-involving motivational climate	− .14	.18*
Coaching harassment experience (rank correlation coefficient)	.03	− .12

*IAT* implicit association test, *p < .05, **p < .01

Next, a multiple regression analysis was conducted to examine the influence of implicit and explicit attitudes on contact behavior and liking that were significantly correlated with IAT scores among the four sports- and exercise-related behaviors (Table 4). The two factors measured usual behaviors related to sports and health; therefore, we expected that they should be explained to some extent by attitudes. The two types of explicit attitudes were measured in both conditions (desirability and joy); however, only the same type of IAT was included in the model because the two explicit attitudes (desirability and joy) were highly correlated. Gender was also included as a predictor variable as explicit (at least) attitudes toward sport and exercise tend to be more positive among male participants.

**Table 4:** Results of multiple regression analysis on behaviors related to sports and exercise

Variable	Desirability IAT cond.	Joy IAT cond.
	β (95% CI)	β (95% CI)
Contact behaviors		
Gender (male = 1, female = 2)	− .15^+^ (− .31 to .01)	− .21** (− .36 to − .05)
Implicit attitudes	.22** (.06 to .38)	.02 (− .13 to .18)
Explicit attitudes	.26** (.09 to .43)	.37** (.22 to .52)
Gender × implicit attitudes	− .06 (− .22 to .10)	.09 (− .06 to .24)
Gender × explicit attitudes	− .06 (− .23 to .10)	− .14^+^ (− .30 to .01)
* R*^2^	.17**	.21**
Liking		
Gender (male = 1, female = 2)	− .05 (− .19 to .10)	− .14* (− .26 to − .01)
Implicit attitudes	.06 (− .08 to .21)	− .05 (− .17 to .08)
Explicit attitudes	.51 ** (.36 to .66)	.62** (.50 to .75)
Gender × implicit attitudes	.05 (− .09 to .20)	.17* (.04 to .30)
Gender × explicit attitudes	.02 (− .13 to .17)	− .10 (− .23 to .03)
* R*^2^	.31**	.45**

*IAT* implicit association test, *cond.* condition, *CI* confidence interval; ^+^*p* < .1, *p < .05, **p < .01

First, we analyzed contact behaviors. In both conditions, higher explicit attitudes were positively related to contact behaviors, namely, the tendency to be exposed to more sports played by celebrities and those covered by the media. In the desirability IAT condition, contact behaviors were more frequent among participants with higher IAT scores. In the joy IAT condition, contact behaviors were significantly more frequent for male participants than for female participants.

Explicit attitudes were also related to liking sports in both conditions. Additionally, an interaction effect between gender and implicit attitudes was observed in the joy IAT condition. A simple slope test revealed that the effect of gender was *β* = − .28, *t*(164) = 3.62, *p* < .001 when IAT scores were − 1*SD*, and the effect of gender was *β* = − .00, *t*(164) = 0.02, *p* = .99 when IAT scores were + 1 *SD*. This indicates that liking is significantly higher in male participants than in female participants when IAT scores are low, whereas there is no gender difference when IAT scores are high.

## Discussion

In this study we measured implicit and explicit attitudes toward sports and exercise, and examined how they are related to the experience of sports and exercise. We assumed that implicit attitudes toward sports and exercise include both cognitive and affective attitudes. The desirability IAT was used to measure implicit cognitive attitudes, and the joy IAT to measure implicit affective attitudes.

First, we explored factors that affect implicit attitudes toward sports and exercise. The male participants displayed significantly more positive implicit attitudes than female participants, for both joy and desirability. It has been repeatedly shown that males favor sports more than females with explicit measures [5, 39], which reflects the fact that sports, having originated in England as a means of education for elite men, are deeply intertwined with the qualities deemed essential for men of that time, and as a result have long been associated with masculinity [40]. This study indicated the same pattern with implicit attitudes.

It was also found that people with sports competence tended to have higher positive implicit attitudes concerning joy, but not desirability. Previous studies reporting positive relationship between competence perceptions and motives for participation in sport argue the enjoyment of sports and physical activities to serve as a mediator for this phenomenon [38, 41]. This study provides empirical evidence for the aspects that previous research had inferred.

Sports experience had little effect on the implicit attitudes toward sports and exercise, except task-involving motivational climate, which had a positive effect on implicit attitudes concerning joy. Hence, Hypothesis 1 was not supported. It is unclear why the effect of sports experience was not observed. With regards to coaching harassment, it is possible that some individuals perceive such experiences as desirable (i.e., helpful, motivated) despite being objectively undesirable [28], potentially offsetting any negative effects. Our participants were Japanese, who are shown to be more positive to coaching harassment than Europeans [42].

Second, the results for the sports/exercise-related behaviors associated with cognitive and affective attitudes were different. Specifically, the degree of recognition of the individual’s physical competence, and their receiving of task-oriented instruction were correlated with the joy IAT only, as was the actual frequency of engaging in sports and exercise. Thus, hypothesis two was supported. This implies that affective implicit attitudes (joy) may be constructed with experience; however, cognitive implicit attitudes (desirability) may be developed in a different way. In addition, positive affective attitudes toward sports and exercise are positively related to current sports/exercise habits, but cognitive attitudes are not.

Contrastingly, desirability IAT scores were positively correlated with ideal exercise frequency and contact behaviors related to professional sports. A multiple regression analysis showed that both implicit and explicit attitudes were associated with contact behaviors, which suggests that contact with sports media and celebrity sports includes both conscious and unconscious attitudes. Based on the dual-process model [21], implicit attitudes lead to unintentional behaviors. We should note that explicit attitudes may be more strongly associated with contact behaviors than implicit attitudes, as the same method is used for measuring the two (self-reports). The strong association between sports/exercise and desirability is related to ideal behavior, while a strong association with joy is related to actual behavior. This result may indicate that a positive affective attitude has the power to overcome obstacles and put ideas into action.

It is interesting to find that male participants with less joyful perceptions of sports and exercise show more liking for sports/exercise and wearing clothes made by sports brands. Although this pattern is difficult to interpret, it may mean that men are more ambivalent about their attitudes toward sports than women. As Smoll and Smith pointed out that boys are encouraged to participate in sports more than girls even in modern times [40]. In other words, there is a prevailing belief that men must have an affinity and aptitude for sports. This is evident in the inclusion of items such as “athletic” and “competitive” in Bem’s measures of masculinity [43]. Under such circumstances, men who do not subconsciously enjoy sports may be more likely to consciously answer that they “like” sports and wear clothes from sports brands. This can also be interpreted in another way. That is, simple clothes from sports brands are suitable as daily wear, and even men who are not good at sports may prefer these clothes because they are familiar and comfortable with them.

Several research problems remain to be solved. First, the standard IAT procedure used in this study is controversial in that it requires an opposing concept in addition to the target concept. We used “sedentary behaviors” as the opposing concept; however, as the stimulus words for this involved indoor hobbies, such as using smartphones and reading, the participants may have responded strongly to this concept instead of to sports and exercise. The IAT scores were calculated relatively and attitudes toward “sedentary behaviors” were also a contributing factor. We need to adopt alternative measurements, such as the single-category IAT developed by Karpinski and Steinman [44].

A positive correlation was found between implicit attitudes, which was measured with IAT scores, and explicit attitudes, which was measured using self-reports. However, the correlation was found to be weak. This pattern is consistent with previous studies [45]. The weak correlation may also be attributed to the IAT structure. As we used paired categories for implicit and unpaired items for explicit indicators, this may be attributed to a discrepancy in the measurement concept. The correlation might have been stronger by using an implicit indicator without a pairwise concept or if a pairwise concept-based measure was used in the explicit indicator as well.

Second, there was only a small difference between the conditions, possibly because the same category labels (positive and negative) were used. More definitive results would have been obtained if not only the stimulus words, but also the category labels were different. Moreover, this study was conducted online. It is possible that the lack of control over the environment in which the participants responded may have influenced the results.

Third, a limitation is that the participants were relatively young and the number of male participants was almost three times that of the female participants. A bias existed as there were more males than females in the classes from which they were recruited. It is possible that characteristic of young men may have affected the results of this study.

To summarize, this study measured two types of implicit attitudes toward sports and exercise, and showed that the sports- and exercise-related behaviors linked to each type are different. The desirability IAT and joy IAT were used to measure cognitive and affective attitudes respectively. It was found that male participants and those with sports competence had more positive implicit attitudes in terms of joy and desirability. Task-involving motivational climate also had a positive effect on implicit attitudes in terms of joy. However, sports experience had little effect on implicit attitudes. Results imply that affective implicit attitudes (joy), not cognitive implicit attitudes (desirability), may be constructed with experience. Positive affective attitudes were found to be related to current sports and exercise habits, while cognitive attitudes were not. We then recommend increasing happy and joyful images of sports and exercise, rather than rational persuasion on the desirability of sports and exercise. For example, sport activities and exercise can be done while happily listening to music in a colorful room, and enjoying delicious treats at the end of activities. The use of such methods may be effective in associating sports and exercise with feelings of enjoyment. Implicit and explicit attitudes were both found to be associated with contact behaviors related to professional sports. Gender differences were also observed. Overall, this study showed attitudes can influence on sports and exercise behaviors.

It is widely understood that sports and exercise are beneficial for the maintenance of health in adults [1, 2]. Furthermore, physical activity promotes brain activity [3] and mental health [4]. In addition, the expansion of human relationships triggered by sports and exercise is expected to have positive effects on the body and mind. We believe that it is meaningful to examine explicit and implicit attitudes related to sports and exercise to help promote lifelong sports participation. Based on the findings of this study, future research can focus on developing interventions aimed at increasing positive affective attitudes toward sports and exercise, particularly among females who showed less positive implicit attitudes compared to males. Additionally, future studies should explore how cognitive implicit attitudes develop and their relationship with sports and exercise behavior. Finally, it is also recommended that interventions aimed at promoting sports and exercise should focus more on creating positive affective experiences rather than relying solely on rational persuasion to promote the desirability of sports and exercise.

## Data Availability

The datasets generated and/or analyzed during this study are available from the corresponding author upon request.

## References

[1] Bize R, Johnson JA, Plotnikoff RC. Physical activity level and health-related quality of life in the general adult population: a systematic review. Prev Med. 2022;2017(45):401–15. 10.1016/j.ypmed.2007.07.017. 10.1016/j.ypmed.2007.07.017 17707498

[2] Moore SC, Patel AV, Matthews CE, et al. Leisure time physical activity of moderate to vigorous intensity and mortality: a large pooled cohort analysis. PLoS Med. 2012;9:e1001335. 10.1371/journal.pmed.1001335. 23139642 10.1371/journal.pmed.1001335 PMC3491006

[3] Soya H, Okamoto M, Matsui T, et al. Brain activation via exercise: exercise conditions leading to neuronal activation and hippocampal neurogenesis. J Exerc Nutr Biochem. 2011;15:1–10. 10.5717/jenb.2011.15.1.1.

[4] Roderick M, Smith A, Potrac P. The sociology of sports work, emotions and mental health: scoping the field and future directions. Sociol Sport J. 2017;34(2):99–107. 10.1123/ssj.2017-0082.

[5] Pedersen MRL, Hansen AF, Elmose-Østerlund K. Motives and barriers related to physical activity and sport across social backgrounds: implications for health promotion. Int J Environ Res Pub Health. 2021;18:5810. 10.3390/ijerph18115810. 34071630 10.3390/ijerph18115810 PMC8198157

[6] Stathi A, Fox KR, McKenna J. Physical activity and dimensions of subjective well-being in older adults. J Aging Phys Act. 2002;10(1):76–92. 10.1123/japa.10.1.76.

[7] Eime RM, Harvey JT, Charity MJ, et al. Age profiles of sport participants. BMC Sports Sci Med Rehabil. 2016;8:6. 10.1186/s13102-016-0031-3. 26973792 10.1186/s13102-016-0031-3 PMC4788892

[8] Kohl HW, Craig CL, Lambert EV, et al. The pandemic of physical inactivity: global action for public health. Lancet. 2012;380:294–305. 10.1016/s0140-6736(12)60898-8. 22818941 10.1016/S0140-6736(12)60898-8

[9] Sims J, Milton K, Foster C, et al. A profile of children’s physical activity data from the 2012 and 2015 health survey for England. BMC Public Health. 2022;22:1785. 10.1186/s12889-022-14150-4. 36127714 10.1186/s12889-022-14150-4 PMC9490976

[10] Japanese Ministry of Health, Labour and Welfare. Summary of National Health and Nutrition Survey 2015. 2016. http://www.mhlw.go.jp/file/04-Houdouhappyou-10904750-Kenkoukyoku-Gantaisakukenkouzoushinka/kekkagaiyou.pdf (in Japanese). Accessed 15 Sept 2022.

[11] Japan Sports Agency. Public Opinion poll on sports practice. 2016. https://www.mext.go.jp/prev_sports/comp/b_menu/other/__icsFiles/afieldfile/2017/02/15/1382031_001.pdf (in Japanese). Accessed 15 Sept 2022.

[12] World Health Organization. Global health risks: mortality and burden of disease attributable to selected major risks. 2009. https://www.who.int/publications/i/item/9789241563871. Accessed 03 Feb 2023.

[13] Trost SG, Owen N, Bauman AE, et al. Correlates of adults’ participation in physical activity: review and update. Med Sci Sport Exerc. 2002;34:1996–2001. 10.1097/00005768-200212000-00020. 10.1097/00005768-200212000-00020 12471307

[14] Weiss MR, Chaumeton N. Motivational orientations in sport. In: Horn TS, editor. Advances in sport psychology. Champaign: Human Kinetics; 1992. p. 61–99.

[15] Van Stralen MM, De Vries H, Mudde AN, et al. Determinants of initiation and maintenance of physical activity among older adults: a literature review. Health Psychol Rev. 2009;3:147–207. 10.1080/17437190903229462.

[16] Eagly AH, Chaiken S. The psychology of attitudes. Fort Worth: Harcourt Brace Jovanovich; 1993.

[17] Hagger MS, Chatzisarantis NLD. First-and higher-order models of attitudes, normative influence, and perceived behavioural control in the theory of planned behaviour. Br J Soc Psychol. 2005;44:513–35. 10.1348/014466604x16219. 16368017 10.1348/014466604X16219

[18] Ohashi MM, Togo E, Iume Y. Difference between sports and exercise in Japanese: a survey on laypeople. Jpn J Appl Psychol. 2022;48:114–5.(in Japanese)

[19] Sasakawa Sports Foundation. The 2017 SSF National Sports-Life Survey of Children and Young People. 2017. https://www.ssf.or.jp/Portals/0/resources/outline/en/pdf/sld2017_kidsteens.pdf Accessed 03 Feb 2023.

[20] Bargh JA, Ferguson MJ. Beyond behaviorism: on the automaticity of higher mental processes. Psychol Bull. 2000;126:925–45. 10.1037/0033-2909.126.6.925. 11107883 10.1037/0033-2909.126.6.925

[21] Bar-Anan Y, Vianello M. A multi-method multi-trait test of the dual-attitude perspective. J Exp Psychol Gen. 2018;147:1264–72. 10.31234/osf.io/bjaq8. 30070579 10.1037/xge0000383

[22] Conroy DE, Hyde AL, Doerksen SE, Ribeiro NF. Implicit attitudes and explicit motivation prospectively predict physical activity. Ann Behav Med. 2010;39:112–8. 10.1007/s12160-010-9161-0. 20140542 10.1007/s12160-010-9161-0

[23] Hyde AL, Elavsky S, Doerksen SE, Conroy DE. The stability of automatic evaluations of physical activity and their relations with physical activity. J Sport Exerc Psychol. 2012;34:715–36. 10.1123/jsep.34.6.715. 23204356 10.1123/jsep.34.6.715

[24] Greenwald AG, McGhee DE, Schwartz JL. Measuring individual differences in implicit cognition: the Implicit Association Test. J Pers Soc Psychol. 1998;74:1464–80. 10.1037/0022-3514.74.6.1464. 9654756 10.1037//0022-3514.74.6.1464

[25] Horn TS. Coaching effectiveness in the sports domain. In: Horn TS, editor. Advances in sport psychology. Champaign: Human Kinetics; 2008. p. 239–67.

[26] Dweck CS. Motivational processes affecting learning. Am Psychol. 1986;41:1040–8. 10.1037/0003-066X.41.10.1040.

[27] Alvarez MS, Balaguer I, Castillo I, Duda JL. The coach-created motivational climate, young athletes’ well-being, and intentions to continue participation. J Clin Sport Psychol. 2012;2012(6):166–79.

[28] Deater-Deckard K, Lansford JE, Dodge KA, Pettit GS, Bates JE. The development of attitudes about physical punishment: an 8-year longitudinal study. J Fam Psychol. 2003;17:351–60. 14562459 10.1037/0893-3200.17.3.351 PMC2755207

[29] Stirling AE. Understanding the use of emotionally abusive coaching practices. Int J Sports Science Coaching. 2013;8:625–39.

[30] Togo E, Iume Y, Ohashi MM. The effect of negative human environment in a team on children’s sports motivation. Jpn J Motiva Stud. 2017;6:17–28. (in Japanese)

[31] Millar MG, Tesser A. Effects of affective and cognitive focus on the attitude–behavior relation. J Pers Soc Psychol. 1986;51:270–6.

[32] Trafimow D, Sheeran P. Some tests of the distinction between cognitive and affective beliefs. J Exp Soc Psychol. 1998;34:378–97. 10.1006/jesp.1998.1356.

[33] Lowe R, Eves F, Carroll D. The influence of affective and instrumental beliefs on exercise intentions and behavior: a longitudinal analysis. J Appl Soc Psychol. 2002;32:1241–52. 10.1111/j.1559-1816.2002.tb01434.x.

[34] Chevance G, Héraud N, Guerrieri A, Rebar A, Boiché J. Measuring implicit attitudes toward physical activity and sedentary behaviors: test-retest reliability of three scoring algorithms of the Implicit Association Test and Single Category-Implicit Association Test. Psychol Sport Exerc. 2017;31:70–8. 10.1016/j.psychsport.2017.11.006.

[35] Greenwald AG, Nosek BA, Banaji MR. Understanding and using the Implicit Association Test: I. An improved scoring algorithm. J Pers Soc Psychol. 2003;85:197–216. 10.1037/0022-3514.85.2.197. 12916565 10.1037/0022-3514.85.2.197

[36] Fujita T, Ebihara M. Personality of sports coach in high school who inflict corporal punishment based on perceived motivational climate: a retrospective approach by university students. Bull Educ Res Dev Faculty Educ Kagoshima Univ. 2014;23:61–6. (in Japanese)

[37] Ohashi MM, Togo E, Iume Y. Exploratory development of a scale to assess coaching harassment experiences in junior sports. Jpn J Appl Psychol. 2022;93:230–9. (in Japanese)

[38] Okazawa Y, Kita M, Suwa Y. Factorial structure of physical competence and its developmental tendency and sex difference. Jpn J Sport Educ Stud. 1996;16:145–55. (in Japanese)

[39] Kaji M, Ono Y. Development of enjoyment scale in physical education classes of elementary school: for higher-grade elementary school pupils. Jpn J Sport Edu. 2020;40(2):1–16. (in Japanese)

[40] Smoll FL, Smith RE. Children and youth in sport: a biopsychosocial perspective. 2nd ed. Dubuque: Kendall Hunt Pub Co.; 2002.

[41] Klint KA, Weiss MR. Perceived competence and motives for participating in youth sports: a test of Harter’s competence motivation theory. J Sport Exerc Psychol. 1987;9:55–65.

[42] Hagiwara H, Wolfson S. Attitudes towards soccer coaches’ use of punishment in Japan and England: a cross-cultural study. Int J Sport Exerc Psychol. 2013;11:57–69.

[43] Bem SL. The measurement of psychological androgyny. J Consult Clinical Psychol. 1974;42:155–62. 4823550

[44] Karpinski A, Steinman RB. The single category Implicit Association Test as a measure of implicit social cognition. J Pers Soc Psycol. 2006;91:16–32. 10.1037/t011832-000. 10.1037/0022-3514.91.1.16 16834477

[45] Greenwald AG, Banaji MR. The implicit revolution: reconceiving the relation between conscious and unconscious. Am Psychol. 2017;72:861. 10.1037/amp0000238. 29283625 10.1037/amp0000238

